# A framework for establishing scientific confidence in new approach methodologies

**DOI:** 10.1007/s00204-022-03365-4

**Published:** 2022-08-20

**Authors:** Anna J. van der Zalm, João Barroso, Patience Browne, Warren Casey, John Gordon, Tala R. Henry, Nicole C. Kleinstreuer, Anna B. Lowit, Monique Perron, Amy J. Clippinger

**Affiliations:** 1 PETA Science Consortium International e.V., Stuttgart, Germany; 2 European Commission, Joint Research Centre (JRC), Ispra, Italy; 3 Organisation for Economic Co-Operation and Development, Hazard Assessment and Pesticides Programmes, Environmental Directorate, Paris, France; 4 National Institutes of Health, Division of the National Toxicology Program, National Institutes of Environmental Health Sciences, Research Triangle Park, NC USA; 5 U.S. Consumer Product Safety Commission, Directorate for Health Sciences, Rockville, MD USA; 6 U.S. Environmental Protection Agency, Office of Pollution Prevention and Toxics, Washington, DC USA; 7 National Toxicology Program Interagency Center for the Evaluation of Alternative Toxicological Methods, Research Triangle Park, NC USA; 8 U.S. Environmental Protection Agency, Office of Pesticide Programs, Washington, DC USA

**Keywords:** Validation, Framework, NAMs, New approach methodologies, Human health, Regulatory

## Abstract

Robust and efficient processes are needed to establish scientific confidence in new approach methodologies (NAMs) if they are to be considered for regulatory applications. NAMs need to be fit for purpose, reliable and, for the assessment of human health effects, provide information relevant to human biology. They must also be independently reviewed and transparently communicated. Ideally, NAM developers should communicate with stakeholders such as regulators and industry to identify the question(s), and specified purpose that the NAM is intended to address, and the context in which it will be used. Assessment of the biological relevance of the NAM should focus on its alignment with human biology, mechanistic understanding, and ability to provide information that leads to health protective decisions, rather than solely comparing NAM-based chemical testing results with those from traditional animal test methods. However, when NAM results are compared to historical animal test results, the variability observed within animal test method results should be used to inform performance benchmarks. Building on previous efforts, this paper proposes a framework comprising five essential elements to establish scientific confidence in NAMs for regulatory use: fitness for purpose, human biological relevance, technical characterization, data integrity and transparency, and independent review. Universal uptake of this framework would facilitate the timely development and use of NAMs by the international community. While this paper focuses on NAMs for assessing human health effects of pesticides and industrial chemicals, many of the suggested elements are expected to apply to other types of chemicals and to ecotoxicological effect assessments.

## Introduction

Data from traditional animal toxicity test methods have been used for many years to inform human health hazard identification and risk assessment. However, studies relying on animals to characterize effects of chemicals can be of questionable or limited biological relevance to human effects (Bracken 2009; Krewski et al. 2010; Leist and Hartung 2013; Hoffmann 2015; Akhtar 2015; Cohen 2017; Paparella et al. 2017; Stewart 2017; Hoffmann et al. 2018; Van Norman 2019; Leenaars et al. 2019).

New approach methodologies (NAMs) are defined as any technology, methodology, approach, or combination that can provide information on chemical hazard and risk assessment, and avoid the use of animals, and may include in silico, in chemico, in vitro, and ex vivo approaches (European Chemicals Agency 2016; U.S. Environmental Protection Agency 2018). NAMs are not necessarily newly developed methods; rather, it is their application to each agency’s regulatory decision-making process or replacement of a traditional testing requirement that is new. This paper focuses on NAMs intended for use in human health hazard identification and risk assessment, but it is expected that what is proposed here also applies to NAMs used for ecotoxicological hazard identification and risk assessment. NAMs can be designed to be relevant to humans, for example, by relying on human cells or dosimetry modeling. They also have the potential to assess key mechanistic events in the development of toxicity, and are intended to be more efficient, relevant, and reliable approaches to risk assessment and regulatory decision making than traditional animal test methods. While NAM development has accelerated rapidly over the last several decades, validation, acceptance, and implementation of these approaches within the context of regulatory decision-making has not kept pace.

A transparent description of the strengths and limitation of both NAMs and traditional animal test methods is needed to address the acceptance and implementation of NAMs (Browne et al. 2019). A number of resources that define the concepts and processes involved in test method validation already exist, notably including the Organisation of Economic Co-operation and Development (OECD) Guidance Document on the Validation and International Acceptance of New or Updated Test Methods for Hazard Assessment (OECD GD 34), which was published in 2005 (OECD 2005). In this paper, we rely on the terminology definitions in the OECD GD 34 (Table 1).

**Table 1:** Definitions of key terms used in this paper as defined by the OECD Guidance Document on the Validation and International Acceptance of New or Updated Test Methods for Hazard Assessment (OECD 2005)

Key term	Definition
Inter-laboratory reproducibility	A measure of the extent to which different qualified laboratories, using the same protocol and testing the same substances, can produce qualitatively and quantitatively similar results. Inter-laboratory reproducibility is determined during the prevalidation and validation processes, and indicates the extent to which a test can be successfully transferred between laboratories, also referred to as between-laboratory reproducibility
Intra-laboratory reproducibility	A determination of the extent that qualified people within the same laboratory can successfully replicate results using a specific protocol at different times. Also referred to as within-laboratory reproducibility
Reference chemicals	Chemicals selected for use in the validation process, for which responses in the in vitro or in vivo reference test system or the species of interest are already known. These chemicals should be representative of the classes of chemicals for which the test method is expected to be used, and should represent the full range of responses that may be expected from the chemicals for which it may be used, from strong, to weak, to negative. Different sets of reference chemicals may be required for the different stages of the validation process, and for different test methods and test uses
Relevance	Description of relationship of the test to the effect of interest and whether it is meaningful and useful for a particular purpose. It is the extent to which the test correctly measures or predicts the biological effect of interest. Relevance incorporates consideration of the accuracy (concordance) of a test method
Reliability	Measures of the extent that a test method can be performed reproducibly within and between laboratories over time, when performed using the same protocol. It is assessed by calculating intra- and inter-laboratory reproducibility and intra-laboratory repeatability
Reproducibility	The agreement among results obtained from testing the same substance using the same test protocol
Test method	A process or procedure used to obtain information on the characteristics of a substance or agent. Toxicological test methods generate information regarding the ability of a substance or agent to produce a specified biological effect under specified conditions. Used interchangeably with “test” and “assay”
Valid test method	A test method considered to have sufficient relevance and reliability for a specific purpose and which is based on scientifically sound principles. A test method is never valid in an absolute sense, but only in relation to a defined purpose
Validation	The process by which the reliability and relevance of a particular approach, method, process or assessment is established for a defined purpose

The underlying principles of validation outlined in OECD GD 34 were developed at an OECD workshop in 1996, “Harmonisation of Validation and Acceptance Criteria for Alternative Toxicological Test Methods”, which aimed to establish scientific confidence in new or updated test methods to support sound science-based regulatory decisions (OECD 2005). While the fundamental principles hold true, there is widespread recognition that the processes used in the past decades for validation need to be updated to encourage timely uptake of fit for purpose and biologically relevant NAMs. The delay between development and regulatory uptake of NAMs is due to many factors, including the failure to clearly define the NAM’s purpose early in the method development process. Additionally, inter-laboratory ring trial studies traditionally used to demonstrate repeatability and reproducibility of animal methods and NAMs are lengthy, expensive, and require coordination of numerous organizations and laboratories. Although the European Union Reference Laboratory for alternatives to animal testing (EURL ECVAM) approach to validation (Hartung et al. 2004) and OECD GD 34 allow for a flexible, modular approach with not all modules being required for all methods, much of the intended flexibility has been lost over time and the validation process has become more rigid and cumbersome than the approach originally envisioned and described in the guidance document. Furthermore, while international regulatory authorities (e.g., at OECD level) have used predictive capacity as a key indication of whether a NAM is relevant and acceptable over the past 20 years, the predictive capacity has usually been determined through comparison to results from traditional animal test methods, for which reproducibility and human biological relevance were often assumed rather than empirically demonstrated. Additionally, many regulatory data requirements were written to be fulfilled by test methods available at the time (i.e., traditional animal test methods). These statutes have complicated gaining regulatory acceptance of NAMs that may not provide identical information to the traditional animal test methods, even if the information provided by the NAMs may in fact be more human relevant and health protective. Such practical limitations must be addressed to establish scientific confidence in, and maximize the use of, NAMs in human health assessments.

An updated framework, designed specifically for establishing scientific confidence in NAMs, should ensure that NAMs are fit for purpose (i.e., fulfil the intended purpose) and provide technically reliable information that is relevant to the understanding of human biology and health protective for the endpoint of concern. Where appropriate and possible, the updated framework may include a demonstration that the NAM provides information of equivalent or better quality and relevance for regulatory decision making as compared, either quantitatively or qualitatively, to the information provided by the traditional animal test method. The process should recognize that the results of the NAM need not directly align with the results of the traditional animal test (Hoffmann et al. 2008, 2018; Kolle et al. 2017; Sewell et al. 2017; Interagency Coordinating Committee on the Validation of Alternative Methods 2018; Piersma et al. 2018; Prior et al. 2019; Clippinger et al. 2021). Additionally, the NAM need not produce the same information generated by the traditional animal test method; in fact, the NAM may be able to provide biologically relevant information and mechanistic insights that are more useful in the regulatory decision making process than the animal test method.

Presented in this paper is a framework to establish scientific confidence in NAMs used to support product registration to inform the regulatory decision making process for human health effects. This framework builds upon criteria developed for evaluating NAMs for skin sensitization that were agreed upon by the International Cooperation on Alternative Test Methods (ICATM) and applied to the validation of the OECD Guideline for the Defined Approaches for Skin Sensitisation (OECD Guideline 497) (Casati et al. 2018; OECD 2021a). While this paper illustrates application of the framework for the assessment of NAMs to predict human health effects for pesticides and industrial chemicals, the framework is expected to be applicable to NAMs developed to measure endpoints of relevance to all chemicals. The use of this framework for evaluating NAMs is expected to increase scientific confidence in, and thus the adoption and uptake of, NAMs across regulatory jurisdictions and chemical sectors.

## A modern, flexible framework to establish scientific confidence in NAMs for the regulatory assessment of chemicals for human health effects

The standard for establishing scientific confidence in a NAM and gaining regulatory acceptance has generally included consideration of whether that NAM can provide information of equivalent or better usefulness, scientific quality, and/or relevance than the existing test method used for regulatory decision making (as appropriate within each agency’s regulatory framework). For example, a criterion in OECD GD 34 for validating any new test method is “the method generates data for risk assessment purposes that are at least as useful as, and preferably better than, those obtained using existing methods. This will give a comparable or better level of protection for human health or the environment” (OECD 2005). More recently, the U.S. Toxic Substances Control Act (amended TSCA; Sect. 4(h)(1)(B)) includes specific considerations for NAMs and mandates that the U.S. Environmental Protection Agency (U.S. EPA) encourage and facilitate the “use of scientifically valid test methods and strategies that reduce or replace the use of vertebrate animals while providing information of equivalent or better scientific quality and relevance that will support regulatory decisions” (15 U.S.C. 2601. 2016). Historically, the concept of “equivalent or better” has relied upon a direct comparison with the traditional animal test data. However, a modern, flexible framework should allow for circumstances in which a comparison to data from traditional animal test methods is not possible or appropriate. For example, for endpoints where no animal test method exists, it is not possible to conduct a comparison. In other cases, the traditional animal test method may be poorly reproducible and, therefore, unreliable as a basis for comparison. In addition, for instances where the traditional animal test method does not provide data relevant to human biology or mechanisms of toxicity, or an analysis of the reproducibility of the animal method is not available to establish performance benchmarks, it may not be appropriate or scientifically justified to compare data from NAMs to animal test method data. Furthermore, NAMs often provide mechanistic information rather than data on apical endpoints measured in animal test methods (e.g., while an observed reduction in body weight in an animal may not provide insights as to the underlying mechanism of toxicity, NAMs may be able to provide these insights). Thus, many new considerations apply to effectively develop a framework to establish scientific confidence in NAMs.

Multiple organizations have published roadmaps to help advance the acceptance and use of NAMs, and there is broad scientific support for developing processes to gain confidence in NAMs. In 2016, the European Chemicals Agency (ECHA) published the proceedings from a scientific workshop, New Approach Methodologies in Regulatory Science, which brought together 300 stakeholders to address the use of information from NAMs to support regulatory decisions for the use of chemicals (European Chemicals Agency 2016). In 2017, the U.S. Food and Drug Administration published a roadmap on advancing NAMs to be used by the FDA’s six product centers (U.S. Food and Drug Administration 2017). Also in 2017, the OECD published guidance for describing non-guideline in vitro test methods (OECD 2017a). In 2018, the 16 U.S. federal agencies that comprised the Interagency Coordinating Committee on the Validation of Alternative Methods (ICCVAM) published a roadmap for establishing NAMs to evaluate the safety of chemicals and medical products (Interagency Coordinating Committee on the Validation of Alternative Methods 2018). Notably, the roadmap identifies the importance of early engagement between regulators and test method developers to ensure NAMs will meet regulatory needs, and encourages agencies to evaluate the relevance of NAMs without relying solely upon data from traditional animal test methods to define performance. In 2021, the U.S. EPA updated its work plan for the development, testing, and application of NAMs that recognized the need for a framework to evaluate the quality, reliability, and relevance of test methods (U.S. Environmental Protection Agency 2021). In 2021, the European Union Reference Laboratory for alternatives to animal testing (EURL ECVAM) surveyed more than 200 stakeholders from academia, industry, government/public sector, and non-governmental organizations, asking how to assess and validate complex in vitro methods, such as 3D cell cultures and organs-on-chips (Joint Research Centre 2021). Participants indicated that these methods should be evaluated for the following four essential qualities for regulatory use: “(a) (human) physiological and biological relevance, (b) reliability (reproducibility, repeatability), (c) predictive capacity, and (d) relevant endpoint” (Joint Research Centre 2021). In 2022, the U.S. Consumer Product Safety Commission published guidance on informational needs for the regulatory evaluation of NAMs (US Consumer Product Safety Commission 2022) and the European Food Safety Authority published a roadmap for action on NAMs in risk assessment (EFSA 2022). These roadmaps contain several common themes, including the importance of defining the regulatory purposes that NAMs are intended to address within the framework of each regulatory agency, communicating with stakeholders, collaborating among public and private groups, providing training on NAMs, establishing confidence in NAMs, and developing metrics for assessing progress on implementation of NAMs.

There has also been substantial work recently to propose evaluation frameworks that support NAMs assessment for specific endpoints. In 2018, ICATM published a list of 12 criteria (adapted from the principles in OECD GD 34) for the evaluation of defined approaches^1^ for assessing skin sensitization (Casati et al 2018). In 2021, the OECD published Guideline 497 on Defined Approaches for Skin Sensitisation (OECD 2021a), in which the defined approaches were reviewed according to an evaluation framework that was adapted from the ICATM paper and was described in an accompanying annex (OECD 2021b). Also in 2018, using the ICATM criteria as a foundation, the U.S. EPA Office of Pollution Prevention and Toxics (OPPT) published a strategic plan to promote the development and implementation of NAMs within the TSCA program (U.S. Environmental Protection Agency 2018). This plan included a list of eight criteria to be used as a starting point for considering scientific reliability and relevance of NAMs used within the TSCA program. The OECD, ICATM, and TSCA frameworks all contain many of the same criteria, including reliability, mechanistic and biological relevance, a defined purpose, and a transparent, independent evaluation of the approach.

Building upon the criteria included in the references above, we propose five essential elements that form the basis of a framework that can be applied by regulators or other end users to establish scientific confidence in NAMs used internationally to assess potential chemical effects on humans and provide information needed for regulatory decision making. Below, we propose the framework, followed by a discussion of each of the essential elements.

### The essential elements of the framework

**Fig. 1:**
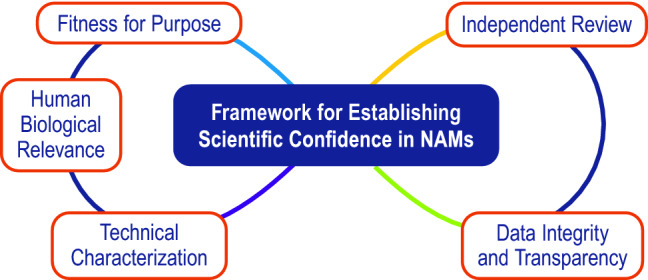
Schematic illustrating the interconnectedness of the five essential elements for establishing scientific confidence in NAMs for assessing human health effects

The proposed framework consists of five essential elements (fitness for purpose, human biological relevance, technical characterization, data integrity and transparency, and independent review) (Fig. 1). The framework is intended to be flexible, including the order of assessment of the elements, which may be addressed separately or in parallel. To establish scientific confidence in a NAM, the process for determining the adequacy of the NAM’s overall performance should be described, including a summary of the fitness for purpose, human biological relevance, and technical characterization of the NAM. The summary, along with supporting information covering data integrity and transparency and independent review, should demonstrate how the various aspects of evidence integrate to support (or oppose) use of the NAM for the intended purpose.

**Fig. 2:**
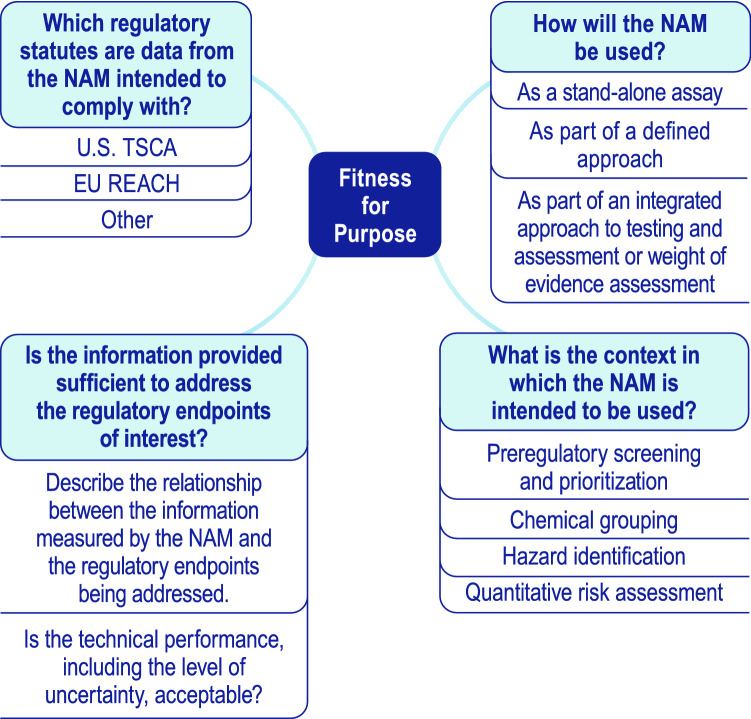
Schematic showing some of the questions relevant to determining the fitness for purpose of a NAM

- Fitness for purpose (Fig. 2)
  - Define which regulatory statute the data from the NAM are intended to comply with (e.g. U.S. TSCA, EU REACH, etc.)
  - Ensure the NAM provides the information that is needed by end-users to come to a conclusion for the chemical under consideration (e.g., qualitative classification, a point of departure, or additional mechanistic information).
    - Define how the information measured by the NAM relates to the regulatory endpoint of interest.
    - Define, qualitatively or quantitatively, the acceptable level of uncertainty for the specified purpose.
  - Define the manner in which the NAM will be incorporated into the assessment (e.g., as a stand-alone assay, as part of a defined approach or integrated approach to testing and assessment/a weight of evidence assessment).
  - Define the context(s) in which the NAM is intended to be used (e.g., for screening/prioritization, chemical grouping, hazard identification, hazard characterization, quantitative risk assessment, etc.)
  - Provide information about the various adverse human health endpoint(s), exposure pathway(s), life stage(s) and population(s) that will be addressed by the NAM.
- Human biological relevance
  - Demonstrate the similarities between the physiology of the test system or the biology measured by the test system, and human biology. Confidence in a NAM is bolstered when it adequately reflects human biological understanding (or, for example, key events in a relevant adverse outcome pathway, AOP).
  - For endpoints where human data or reference chemicals are available, demonstrate concordance of the NAM with human responses to build confidence in its human biological relevance.
  - When applicable, evaluate the traditional animal test method(s) in either a quantitative or qualitative capacity, taking into account the human biological relevance. When comparisons are appropriate, demonstrate that the NAM reflects human biological understanding as well as or better than the traditional animal test method.
- Technical characterization
  - Evaluate the protocol, the equipment used, and any computational models being used for endpoint prediction and/or in vitro to in vivo extrapolation.
  - As outlined in OECD GD 34, assess and describe the intra-laboratory reproducibility, transferability (where applicable), applicability domain, associated reference chemicals and controls, and limits of detection and quantification.
  - Where relevant, assess and describe the accuracy of the NAM. Evaluation of accuracy through comparisons to data from the traditional animal test method should not be the default; however, where data from the traditional animal test method are available, it may be useful to determine performance benchmarks for the NAM on the basis of the reproducibility of the animal test method data.

- Data integrity and transparency
  - Demonstrate the integrity and credibility of the data submitted for assessment and peer review (from the raw data to the final report).
  - Communicate transparently and, as far as possible, make publicly available information about a NAM’s relevance to human biology, fitness for purpose, and technical characterization, as well as the principles of the NAM, the protocol, and reporting standards.
  - Assess and describe the uncertainties and limitations associated with the NAM.
- Independent review
  - Determine the appropriate level of external review necessary for a NAM. Peer review and publication of a NAM’s human biological relevance, fitness for purpose, and technical characterization is encouraged. In certain cases (e.g., for novel applications), NAMs may be reviewed by independent third parties.

## Discussion of the essential elements

The following discussion expands on the essential elements of the proposed framework.

### Fitness for purpose

A NAM that is fit for purpose is suitable and useful for its intended purpose, particularly for informing risk assessment and management decisions (U.S. Environmental Protection Agency 2014). A fit for purpose NAM provides information that assists end-users in drawing a conclusion for the chemical and endpoint under consideration (alone or in combination with other data). The type of information provided by the NAM should align with the type of information required or needed, and with the level of acceptable uncertainty defined by the regulators or other end-users for a specific purpose (U.S. Environmental Protection Agency 2021). To determine whether a NAM is fit for purpose, one would therefore evaluate whether the information provided by the NAM addresses the points listed under the “Fitness for purpose” section of the framework.

Preferably, before a NAM is developed, its purpose should be clearly defined and discussed among the method developer, regulators, and the regulated industry. It is not necessary for the NAM to be a one-to-one replacement for the traditional animal test method, provide identical information to the traditional animal test method, nor to predict the exact results of the animal test method; rather, it must provide key information needed to help make a health protective regulatory decision. The NAM does not necessarily have to be an OECD or regulatory agency test guideline to fulfil its intended purpose.

Under TSCA, the U.S. EPA OPPT encourages applicants with new industrial chemicals to attend “pre-notice consultations” before conducting any testing to ensure that the testing strategy proposed by the applicant is likely to provide sufficient information for the regulators to perform risk assessments and make regulatory decisions. Similarly, the U.S. EPA OPP encourages pesticide registrants to meet with the agency to discuss the use of NAMs. In the EU, plant protection product and active ingredient applicants are encouraged to meet with EU member state regulatory authorities and/or the European Food Safety Authority. These meetings provide applicants and regulators opportunities to maximally integrate NAMs for defined purposes within the assessment of industrial chemicals and pesticides. Additionally, formal regulatory programs exist to facilitate collaboration and early discussions between regulators, including the Accelerating the Pace of Chemical Risk Assessment (APCRA) Initiative, and between regulators and stakeholders for other chemical classes, including the U.S. Food and Drug Administration’s Medical Device Development Tools program (for medical devices) and the Innovative Science and Technology Approaches for New Drugs (ISTAND) program (for pharmaceutical drugs).

### Human biological relevance

In the case of human health, biological relevance is determined by demonstrating similarities between the physiology of the test system used or the biology measured by the test system and human biology. Scientific confidence is increased when the NAM captures key aspects of human biological information as well as or better than the traditional animal test method, and provides information that allows regulators to make decisions that protect human health. Establishing biological relevance of NAMs offers an alternative to benchmarking the performance of the NAM using results from traditional animal test methods. Given the limitations inherent to interspecies extrapolation, emphasis should be placed on human biology as the basis for NAM development and assessment.

#### Human biological information

Human biological information incorporates an understanding of human biology as well as mechanisms of toxicity that are relevant to human biology. Relevance to human biology can be demonstrated by, for example, incorporating information from human dosimetry modeling, or by evaluating the cell type(s)/tissues used in the NAM, the structure of the target organ/tissue, or the presence of human-relevant metabolites associated with the parent chemical tested (noting that metabolites found in humans might be different from those found in animals or produced in a NAM) (Browne et al. 2019).

In addition to biology, one should consider the ability of a NAM to assess mechanism(s) of toxicity (Hartung 2010; 2013; Parish et al. 2020; Madia et al. 2021). For example, one might consider an AOP, which is an organizational construct that relates chemical interactions with molecular initiating events, leading to downstream cellular and tissue-level effects, which prompt adverse outcomes in organisms and across populations (OECD 2021c). AOPs can be useful for organizing data collected in independent experiments and measuring effects at different levels of biological organization to evaluate relationships between key events in a toxicity pathway. NAMs may measure mechanistic and cellular effects early in the pathway and, thus, predict downstream effects. In cases where an AOP is available, the NAM does not have to cover all key events within the AOP to be useful. For skin sensitization, data requirements within the European Union regulations of chemicals (REACH) and biocides (BPR) request the use of NAMs that supply information on key events within the well-defined skin sensitization AOP (European Commission 2019, 2021). In the absence of an existing AOP, mechanistic information can be collected from the scientific literature (e.g., using systematic reviews) or generated in vitro.

There are multiple cases where the test species was determined not to be an appropriate model for human biology. The ability of in vitro, ex vivo, and traditional in vivo rabbit test methods to assess human-relevant biology and mechanisms of eye irritation/corrosion have been evaluated (Clippinger et al. 2021). To provide historical context, the in vivo rabbit test method for assessing eye irritation and corrosion of chemicals was developed in 1944 and adopted as an OECD test guideline in 1981 without undergoing a formal validation process (Draize et al. 1944; OECD 2017b). The review paper examined the structure of the eye in humans and other species used in in vitro, ex vivo, or in vivo eye testing, and the mechanisms that lead to eye irritation in humans. A range of NAMs, and the traditional animal test method, were analyzed based on the coverage of human biology and mechanistic relevance assessing the depth and degree of injury in the human eye. The reliability (inter- and intra-laboratory reproducibility) and cell types/tissues (species and types of cells) used in each method were also discussed. Based on the information presented, the authors concluded that the rabbit test method is not a valid method to which NAMs should be compared and NAMs should be evaluated by determining the biological relevance of the test system to human biology. While the paper focused on eye irritation, the approach used in Clippinger et al. (2021) could be used to evaluate many different endpoints.

A similar approach has been considered for respiratory toxicity testing (Clippinger et al. 2018). Upon review of the anatomy and physiology of the human and rodent respiratory tracts, clearly there are numerous differences that affect deposition and clearance of substances and, therefore, the results of toxicity test methods. For example, between rodents and humans, there are differences in the branching pattern of the respiratory tract, in the expression of key genes, and in mucous secretion (Kolanjiyil et al. 2019). These key differences underscore the importance of understanding human biology to determine the biological relevance of a toxicity test method.

As another example, it is well recognized that traditional developmental neurotoxicity (DNT) animal test methods do not provide reproducible and easily interpretable information on the developing brain that is suitable for regulators (Tsuji and Crofton 2012; Smirnova et al. 2014). The windows of susceptibility and processes of fetal brain development differ between humans and other animals, and interpretation of “adversity” as an endpoint is particularly challenging in the traditional DNT animal test methods. Thus, NAMs that use human relevant test systems may better account for the biological processes and be more easily interpreted than the lengthy and expensive animal test methods. Consequently, in 2018, a new process for assessing DNT NAMs was proposed. An international collaboration of scientists, regulators, and stakeholders, including OECD, the U.S. EPA, and the European Food Safety Authority, have worked on a strategy to develop a battery of in vitro assays that evaluate critical processes of neurodevelopment that can overcome the limitations of the in vivo DNT studies (Bal-Price et al. 2018a, b). The OECD is developing a guidance document to aid in the evaluation of the battery of in vitro assays and, most recently, the U.S EPA Office of Research and Development used the battery in a weight of evidence assessment to support a DNT waiver for a specific chemical (Dobreniecki et al. 2022).

#### Human in vivo data

For endpoints used to protect human health, the ultimate reference data are high quality human data. However, for most endpoints and chemicals, human data are sparse, unavailable, or of poor quality. Where high quality human data are available, the ability of NAM(s) to accurately predict the human response should be as good as or better than the accuracy of the traditional animal test method to predict the same human data, where comparison is appropriate and possible.

High-quality human epidemiological data can be difficult to obtain and align with toxicological data. When high quality epidemiology data are available, they may be useful in identifying environmental chemicals with associations to adverse human health effects. When the human data available are not of sufficient quality to be used alone, they may be used in combination with other existing data to establish scientific confidence in NAMs (Krishna et al. 2021).

Data from studies of human volunteers are available for some endpoints, such as skin sensitization. Hazard and potency results for reference chemicals obtained from defined approaches for skin sensitization were compared to curated human and local lymph node assay (LLNA) data (Hoffmann et al. 2018; Kleinstreuer et al. 2018). It was demonstrated that the defined approaches, comprised of in silico, in chemico, and/or in vitro models, were more predictive (76–85%) of the human hazard data than the mouse-based LLNA (74%) when predicting whether or not a chemical will cause skin sensitization. The results also showed that, for predicting three human potency classes, the non-animal defined approaches performed at 55 percent to 69 percent whereas the mouse-based LLNA was predictive 59 percent of the time. OECD Guideline 497 was the first guideline to include a comprehensive curation of human reference data set to support the demonstration that defined approaches were superior to the animal method when compared to the human data (OECD 2021a).

### Technical characterization

Technical characterization of NAMs is conducted to ensure a NAM is high quality and robust. Below, we examine relevant considerations of technical characterization, namely the evaluation of accuracy, reproducibility, transferability (where applicable), applicability domain, associated reference chemicals and controls, and limits of detection and quantification. Some of these points have been recently discussed for the assessment of inhalation toxicity NAMs in Petersen et al (2021a, b) and, more recently and with a broader applicability, in Petersen et al. (2022). The authors published a framework to add measurement quality features to in vitro and in chemico NAMs (including conceptual evaluation of the assay, intra-laboratory evaluation, statistical data analysis and reporting, and, on a case-by-case basis, inter-laboratory evaluation), to ensure that sources of variability are identified and mitigated (Petersen et al. 2022).

Specific aspects of technical characterization will depend on the NAM being evaluated and its intended use. For example, increased uncertainty or performance variability, or qualitative results, may be acceptable for NAMs intended for screening purposes compared to those intended for use in quantitative risk assessment. The OECD Guidance Document on Good In Vitro Method Practices (OECD GD on GIVIMP) provides a comprehensive introduction to the elements of technical characterization that should be described (OECD 2018). It should be demonstrated that the data have been generated using appropriate assays for the question at hand, and the data reporting should allow for agencies or organizations to evaluate the NAM, the protocol, and the equipment used, and should properly describe any computational models being used for endpoint prediction and/or in vitro to in vivo extrapolation.

#### Accuracy

Accuracy is the measurement of how closely the test method results agree with accepted reference values. While accuracy has historically been determined by comparing NAMs to the traditional animal test methods, this should not be the default. Test methods using animals are often decades old and were accepted as OECD test guidelines without undergoing a rigorous validation for relevance or reproducibility.

Recent publications have assessed the results of traditional animal test methods and have demonstrated a low level of reproducibility for a range of endpoints, including the LD_50_ test in rats for acute oral toxicity (Karmaus et al. 2022), Draize rabbit tests for eye irritation (Weil and Scala 1971; Adriaens et al. 2014; Luechtefeld et al. 2016; Barroso et al. 2017), and skin irritation (Rooney et al. 2021), the LLNA using mice for skin sensitization (Dumont et al. 2016), the Uterotropic assay using rodents for estrogen receptor testing (Kleinstreuer et al. 2016), the Hershberger assays using rodents for androgen receptor testing (Browne et al. 2018), rodent cancer bioassays (Paparella et al. 2017), and repeat-dose toxicity studies (Pham et al. 2020).

Where traditional animal test methods do not provide data relevant to human biology or mechanisms of toxicity, a comparison of that data with data from NAMs may not be appropriate. Additionally, for endpoints that have not traditionally been assessed using animals, and for which animal test methods do not exist, clearly, the comparison is not possible. However, when evaluating the accuracy of the NAM through comparisons to data from traditional animal test methods, several factors should be considered:

- The reproducibility of the traditional animal test method represents the level of reproducibility that regulators currently accept when using such data to conduct safety assessments and a similar bar should be acceptable for NAMs.
- Where results from the animal test method and the NAM conflict, the reproducibility of the animal test method, when known, as well as its relevance to human biological information could explain the differences observed.
- Understanding the reproducibility of, and reasons for observed variance in, the traditional animal test method allow for setting realistic expectations about the capabilities of the animal method, and therefore, the maximum performance capacity of NAMs that are compared against them (Browne et al. 2019). The ability of the NAM to predict the result of the traditional animal test method should not be expected to exceed the ability of the animal test to predict itself (i.e., the accuracy/predictive capacity of NAMs should not be expected to exceed the reproducibility of the animal test method).
- Traditional animal test method reproducibility can be used to derive confidence intervals that provide performance benchmarks for NAMs. If a NAM is compared to a traditional animal test method result, the NAM prediction falling within the data-driven confidence interval around the animal result would be considered to be concordant with the animal result. When the reference test method is demonstrated to have a low degree of reproducibility, the category assignments based on those results may be close to arbitrary for some chemicals.
- When the accuracy of a NAM is determined by a comparison to existing data, it should be clarified whether it was compared to human (the gold standard) data or animal data.

Use of publicly accessible curated databases allows for further assessments of reproducibility of traditional animal test methods, including the evaluation of statistical variability and the limits of detection (Browne et al. 2015; Mansouri et al. 2016, 2020; Barroso et al. 2017; Judson et al. 2018a; Karmaus et al. 2022). Manual curation of data can be time-consuming but allows curators to identify errors common in reporting (e.g., misplaced decimal points or incorrect units), and quantitative confidence intervals can be incorporated into in silico tools (Karmaus et al. 2022). While curation of data may improve reproducibility, it will not increase the biological relevance to human health effects.

To provide additional confidence, accuracy can be evaluated by determining the NAM’s ability to correctly identify positive and negative reference chemicals for the human health endpoint of concern identified based on a weight of evidence of multiple data (where applicable). Alternatively, accuracy can be evaluated by making use of internal/external consistency (Patterson et al. 2021) also known as “orthogonal validation”. In other words, it can be useful to compare and combine data obtained with different NAMs that are relevant to the same endpoint, key event, and/or pathway. Efforts related to the estrogen receptor and androgen receptor pathways demonstrated such an approach in support of the U.S. EPA Endocrine Disruptor Screening Program (Browne et al. 2015; Judson et al. 2015; Kleinstreuer et al. 2017). When multiple NAMs relevant for the same mechanistic events/pathways confirm each other’s results, this increases confidence that the results obtained are relevant.

#### Reproducibility and transferability

Reproducibility is the measurement of how closely results of a particular method agree when repeated using the same protocol and same substance (OECD 2005). To assess the reproducibility of a method, it may not always be necessary to perform an exhaustive assessment of inter-laboratory reproducibility, i.e., a ring trial. It may be preferable, depending on the NAM and whether the NAM will be performed in more than one location, to assess reproducibility solely through the conduct of intra-laboratory reproducibility testing. The intra-laboratory reproducibility of a NAM should not be expected to exceed the reproducibility of the traditional animal test method, when the reproducibility of the animal method is known. While the reproducibility of the animal test method may be useful in identifying performance benchmarks for the NAM when the data exist, the advancement of NAMs should not be delayed if the reproducibility of the animal test method is unknown.

For NAMs that will be performed in more than one location, properly designed training and transfer studies in a second independent and competent laboratory using a small number of coded chemicals, should be conducted to show that the NAM can be repeated in a naïve laboratory (Hartung et al. 2004). In combination with a robust assessment of intra-laboratory reproducibility, training and transferability of the method to a second laboratory aids reliability by providing an opportunity to improve clarity and verify the independent implementation of the protocol. Moreover, post-validation proficiency testing adds confidence on the capacity of a laboratory to use the test method. Performance-based validation relies upon a set of robust reference chemicals with biologically relevant activities against the target/pathway of interest. This may be a more effective and efficient option, depending on the NAM’s purpose, than a costly and lengthy ring trial to demonstrate inter-laboratory reproducibility. Furthermore, an exhaustive assessment of inter-laboratory reproducibility, such as is performed during ring trials, may reflect laboratory quality or expertise rather than a NAM’s reliability.

#### Applicability domain

The applicability domain describes the physicochemical or other properties of the substances for which a particular method is applicable for use (OECD 2005), although it is often more practical to describe the properties of substances for which the method is not applicable (i.e., classes of chemicals that would interfere with the method or which the method cannot accurately measure—technical, mechanistic and predictive limitations). NAMs should be considered generally acceptable for a wide range of chemical classes, functional groups, single ingredients, and mixtures unless there is evidence that a particular chemistry would interfere with the integrity of the assay or not be mechanistically covered considering the test system used and endpoint measured. The OECD GD on GIVIMP provides a non-exhaustive list of physical, chemical, and other properties that should be reported in the description of the applicability domain (OECD 2018). The list includes the state of the substance, its appearance and size, its color, pH and other physicochemical properties, composition, conditions of stability, expiry date, and solubility.

#### Reference chemicals and controls

Positive control reference chemicals are chemicals that have been demonstrated to be causally linked to an adverse effect(s) in humans, whereas negative control reference chemicals have been demonstrated to not adversely affect humans, for a particular endpoint (OECD 2005). Positive assay controls, on the other hand, are chemicals that reliably induce an adequate response in the test method, and negative assay controls reliably do not. Characteristics to consider when choosing reference chemicals (Kolle et al. 2019) as well as assay positive controls (Petersen et al. 2021a) have been published.

Selection of reference chemicals should take into account the purpose of the NAM, and one definitive list may not cover all purposes. The formal validation process outlined by the OECD encourages testing a range of reference chemicals; however, the compilation of reliable reference chemicals is time consuming, resource intensive, and some substances can present storage and stability challenges. Furthermore, it can be difficult to compile a robust reference chemical list that covers a broad chemical space. Additionally, an emphasis within reference sets on positive reference chemicals can lead to high false positive rates; therefore, reference sets should have a balance of positive and negative control reference chemicals, when possible (Browne et al. 2019). In circumstances where a comprehensive list of reference chemicals is not available for a particular endpoint, for example, for novel endpoints, the emphasis should instead be on the identification of appropriate positive and negative controls for use during the evaluation of the individual assays with consideration of the NAM’s applicability domain and purpose.

The hazard properties of reference chemicals for a certain toxicological endpoint should be established based on a weight of evidence evaluation of all available data for each chemical, including (human and animal) in vivo, in vitro, in silico endpoint and mechanistic data, rather than establishing such properties on the basis of individual traditional animal test methods because of the known variability and uncertainty associated with such studies. The EURL ECVAM library of reference chemicals is a catalogue of chemical lists that can be used to standardize, qualify, characterize or compare in vitro, in chemico and in silico methods and models (Sund and Deceuninck 2021). It contains chemical lists used in research and validation projects (including EU-funded, international and JRC projects), proficiency chemicals from OECD test guidelines, and chemicals that have been classified within various regulatory contexts (e.g. pesticides, carcinogenic chemicals, and endocrine disrupters). Curated reference chemicals lists, compiled via rigorous systematic reviews and automation processes where feasible, have allowed more robust evaluation of NAM sensitivity and specificity (Judson et al. 2018b).

#### Limits of detection and quantification

The limit of detection defines the lowest concentration or signal of a substance that can be distinguished, with acceptable precision, from the absence of that substance (OECD 2018). Integral to the assessment of technical characterization is the description of the equipment used in the NAM and the limits of the instrument readouts available. The dynamic range (the ratio of maximum and minimum concentrations over which the parameter being measured can be detected by the instrument used in the NAM), or more specifically, the linear dynamic range (the range of concentrations over which the instrument read-out for the measured property is linear) should be described, where appropriate. The lower limit of the linear dynamic range is often refered to as the lower limit of quantification and the higher limit of the linear dynamic range is often refered to as the higher limit of quantification (OECD 2018).

Limits of detection cannot be calculated for qualitative methods. For qualitative methods, the minimum concentration of substance that can be detected within a specific probability of error, i.e., a cut-off value, should be calculated instead (OECD 2018). The cut-off values are often established by assessing the rate of false positive and negative values above and below the expected cut-off concentration.

### Data integrity and transparency

Transparency facilitates trust in the use of NAMs and thereby hastens the pace of each agency’s regulatory decision-making process and potential regulatory acceptance. A NAM’s relevance to humans, fitness for purpose, and technical characterization should be transparently communicated to peer reviewers, the scientific community, and to the public in a manner digestible by non-scientists. Where appropriate, peer reviewed articles and information describing the fitness for purpose, biological relevance, and technical characterization of the NAM should be published in open-access journals and/or summarized in public-facing regulatory documents. In addition, where possible, the principles of the NAM, the protocol, and the reporting standards should be communicated publicly. For NAMs that contain intellectual property, the OECD provide tools to maintain transparency, including reasonable and non-discriminatory terms (“RAND”) for licensing commitments (OECD 2021d). ICATM partners publish information on NAM assessment and peer review via the Tracking System for Alternative methods towards Regulatory acceptance (TSAR) (European Union Laboratory for Alternatives to Animal Testing 2021). TSAR indicates the stages NAMs have reached in terms of acceptance as a recognized standard for use in a regulatory context together with a summary description and accepted protocol(s)/SOP(s). Where available, TSAR also includes relevant records and documents associated with a NAM linked to the different steps of the entire process: submission, validation, peer-review, recommendations and regulatory acceptance. How to interpret the data that a NAM generates should be clearly communicated so that end-users understand the process and can apply it in a practical setting. Additionally, if NAMs undergo an independent scientific review by third parties, this review should be made publicly available.

It is advisable that method developers organize an independent evaluation of the processes used for the acquisition, transferring, and processing of raw data before those data are submitted to external, independent parties for assessment and peer review to ensure data integrity and credibility of results. Studies should be conducted to the extent possible in the spirit of the principles of good laboratory practice. Furthermore, evaluating bodies, such as the U.S. NTP Interagency Center for the Evaluation of Alternative Toxicological Methods can fund assessments of the quality and integrity of the development process of the NAM. For in vitro methods, developers can also consult the OECD GD on GIVIMP (OECD 2018) to reduce uncertainties in predictions. For digital tools, and management of digital data, developers can follow the ‘FAIR Guiding Principles for scientific data management and stewardship,’ published in 2016 (Go Fair 2016).

#### Uncertainties

Uncertainty refers to all types of limitations on the available knowledge that may affect the range and probability of possible answers to an assessment question (Benford et al. 2018). Uncertainties related to the data and methodological quality include limitations associated with the method’s biological relevance, reproducibility, and completeness or comprehensiveness of the data (OECD 2020). To minimize these uncertainties, guidance on GIVIMP has been developed by the OECD (OECD 2018) to demonstrate best practices for execution and reporting. Other uncertainties are associated with the interpretation, extrapolation, and integration of data from the test method, and includes inter- or intra-species uncertainty factors, in vitro to human extrapolation, and gaps in knowledge of the endpoint of concern.

During the evaluation of the NAM, the uncertainties of the NAM and the traditional animal test method, where appropriate and possible, should be clearly and concisely described. More important than a comparison to the animal test method is evaluating the NAM’s fitness for purpose, relevance to human biology, and technical characterization. Furthermore, depending on the purpose of the NAM, for example whether the data from the NAM will be used alone, in combination with other information sources in a defined approach, or as part of an integrated approach to testing and assessment or a weight of evidence assessment, the acceptable level of uncertainty associated with the NAM may vary.

### Independent review

Information and data supporting the NAM’s fitness for purpose, human biological relevance, and technical characterization may be scientifically reviewed by independent third parties (whose members do not have a conflict(s) of interest); however, the necessary level of review will be identified by each agency’s decision makers.

Raw data from, and information describing, the NAM should be accessible for review by independent third parties and regulatory agency decision makers. The assessment and independent peer review of NAMs may be organized by validation bodies, such as EURL ECVAM and its Scientific Advisory Committee (ESAC) or the Japanese Center for the Validation of Alternative Methods (JaCVAM). Other bodies or international organizations that can independently review NAMs include the U.S. Federal Insecticide, Fungicide, and Rodenticide Act Scientific Advisory Panel, the European Food Safety Authority Scientific Committee and the OECD. Alternatively, the developer can fund (but not directly manage) an independent review of the method. Peer-reviewed publications are useful for sharing assay information with the scientific community and can supplement a more formal review by independent third parties to gain acceptance and use of the method in a regulatory context.

## Conclusion

The proposed framework is comprised of the following five elements: fitness for purpose, human biological relevance, technical characterization, data integrity and transparency, and independent review. Use of the framework will enable a streamlined confidence building process that allows for the timely uptake of fit for purpose and biologically relevant NAMs that can be used in the regulatory decision making process as appropriate within each agency’s regulatory framework. Instead of relying on a direct comparison to the currently used animal test method, the framework encourages a holistic assessment of a chemical’s ability to cause toxicity in humans by relying on NAMs that reflect human biological understanding. Use of the framework should provide better coverage of human biology and mechanisms of toxicity, increase confidence in the appropriate use of NAMs, and accelerate industry uptake and regulatory acceptance of relevant and reliable NAMs, thereby providing better protection of human health.

## Footnotes

1 A defined approach consists of a fixed data interpretation procedure (e.g., a mathematical model or a rule-based approach) applied to data (e.g., in silico, in chemico, or in vitro data) generated with a defined set of information sources to derive a prediction without the need for expert judgment (OECD 2021a).
